# Chronic Constipation

**Published:** 1906-05

**Authors:** L. Amster

**Affiliations:** Atlanta, Ga., Physician to Wesley Memorial Hospital; Piedmont Sanatorium; Hebrew Orphans’ Home


### CHRONIC CONSTIPATION.*

BY L. AMSTER, M.D., ATLANTA, GA.,

Physician to Wesley Memorial Hospital; Piedmont Sanatorium; Hebrew Orphans' Home.

We may probably reap no scientific laurels from the study of chronic constipation. We may consider this ailment a trivial, though disagreeable affliction, not worthy of careful attention, when the large numbers of laxative and purgative remedies at our command will relieve the condition, p. r. n. We may shirk the responsibility and not subject ourselves and our patients to a test of patience and perseverance in the treatment. But what happens?

Eventually our patients turn from us to the muchly lauded, advertised nostrums, or we have to suffer the mortification that our patients are cured by osteopaths, nature healers, etc.

The number of sufferers are legion; they rightfully belong to us, and have a right to expect permanent benefit from our skill. We can do this better and safer than the charlatans, if we only take the trouble.

In the whole realm of medicine, there is no condition that can be as successfully overcome as that of chronic constipation. The opportunity of making grateful friends of our patients is unbounded.

This has been my experience and the experience of every physician who has given more than superficial attention to the subject.

Under habitual constipation we understand a condition by which the intestines empty their contents in an irregular and incomplete way. This definition does not refer to cases that show no ill effects and no disturbance of personal well-being by virtue of retarded defecation. We know that there are people who are perfectly well and normal, but who have but one bowel movement every other day or every two days. We may term such a condi-

*Read before the Fulton County Medical Society.

tion "physiologic constipation." On the same order we observe tachycardia, a physiological quickening of the heart action. Just like there are women who do not menstruate but every two or three months under normal conditions.

*Idiopathic,* habitual constipation produces a certain train of symptoms which disturb the well-being more or less.

### CAUSES OF HABITUAL CONSTIPATION.

Before enumerating the causes of habitual constipation, we have to consider a group of cases without any etiological factor. The malady develops from some insignificant, trivial cause, the peristalsis is slightly impaired, and at first the patient does not pay any material attention to it, occasionally resorting to some purgative or enema. Gradually purgative remedies have to be increased, and so it continues from year to year until the stronger remedies become ineffectual. Finally, discomfort, pain, or catarrhal manifestations remind the patient that it is about time to employ some fixed, rational treatment.

Often we can trace habitual constipation to hereditary influences. It seems to involve whole families to a more or less degree. Initial symptoms often appear in early infancy and are somewhat troublesome to overcome.

Occasionally this hereditary tendency is associated with a lack of proper management of children.

Children are not taught the importance of going regularly to the stool. Some children, girls especially, do not respond to nature's call at school either from false modesty or fear of the teacher.

A very frequent cause of habitual constipation is the irrational choice of food. Boas designates this condition as "alimentary constipation." Overindulgence in meats and lack of vegetables and fruits, i. e., foods which contain a large amount of residue, thereby stimulating peristaltic action.

Sometimes patients acquire alimentary constipation (by the physician's advice) through rigid dietary adherence in diabetes, or through milk cures in chlorosis and nephritis.

The origin of some cases of constipation we can trace to medicinal causes. It often occurs in cases of acute gastro-intestinal disturbances, like entero-colitis, peritonitis, appendicitis, etc., where large doses of opium or belladonna are, I may say, wrongfully employed, resulting in severe, often irreparable intestinal paresis. Too liberal use of tannic acid preparations in diarrhea often result in constipation.

A more frequent cause of constipation is the lack of exercise. Still, this cause is often overrated, as it is generally known that, e. g., letter-carriers, farmers, bicycle-riders, etc., are not exempt from the ailment.

One of the most important etiological factors of chronic constipation is neurasthenia or an intestinal neurosis. Patients suffering from enteroptosis, the majority of which are also neurasthenic and hysterical, are almost always habitually constipated.

Finally, we may say that irregularities in the mode of living, irregular work, irregular eating, irregular sleep, and consequently irregular defecation, are important etiological factors in causing habitual constipation.

### SYMPTOMATOLOGY.

The symptoms vary according to degree and variety of constipation.

As to degree:

(a) The large intestines are weakened in their muscular activity either in toto or in part (especially the upper segment).

(b) The intestinal contents may be propelled normally—stagnation occurring in the sigmoid flexture of rectum, i. e., the inability of expulsion rests in the last portion.

As to variety, Fleiner distinguishes clinically two varieties:

1. The atonic form.
2. The spastic form.

The *atonic* form constitutes simple atony or weakness of the intestine due to faulty nutrition and irregular mode of living. The stool is harder than normal and of large caliber.

The *spastic* form is the constipation of irritable neurasthenics

—hypochondriacs and women with splanchnoptosis. The stool appears as narrow, cylindrical bands or as small round balls of hazel-nut size. The formation is due to contraction of intestinal coils.

Both forms may consist or be accompanied by catarrhal condition of the intestines.

Boas describes a third form, which he names "fragmentary constipation"—regular, spontaneous, but incomplete movements. The patient has to go to stool every two or three hours and after much effort, accompanied by a sense of fulness, pressure and tenesmus, manages to evacuate small fragments or a mushy mass. This trouble seems caused by a torpidity of the lower portion of the large intestine and rectum.

Two prominent symptoms of habitual constipation, especially of the spastic form, are *colicky pain* often accompanied by *flatulency,* both very annoying and usually relieved after copious defecation.

Bouchard claims that the headache, neuralgia, dizziness, mental depression and malaise are due to autointoxication. Some clinicians, however, claim that this theory can not be substantiated in a large number of cases, as it is possible to cure these neurasthenic patients of their constipation without getting rid of the above-mentioned symptoms supposedly due to autointoxication.

Some few cases of chronic constipation show albuminuria with cylindruria, both of which disappear after the constipation is relieved. A hasty diagnosis of Bright's disease in these cases, therefore, is not justified.

### DIAGNOSIS.

At first glance, it would seem that the diagnosis of habitual constipation is very simple, and indeed such is the case in the large majority of cases. But, unless we subject our patients to a close general and local examination, we are apt to fail in our attempt at permanent relief. We must convince ourselves that the condition is not due to piles, tumors, fissures of the rectum, to prostatic hypertrophy, ovarian and uterine tumors, retroflection, benign and malignant stenosis.

TREATMENT.

It is understood that the treatment refers to primary, habitual constipation.

The success of the treatment depends largely upon the recognition as to what form of constipation we have to deal with, either atonic or spastic. As a rule, cases that did not have to resort in the past to the more drastic purgatives yield better results, regardless of the length of time of the affliction.

The treatment may be divided:

(a) Prophylactic.
(b) Dietetic.
(c) Mechanical.
(d) Electric.
(e) Hydrotherapeutic.
(f) Medicinal.

1. The prophylactic treatment consists in the proper management of childhood as to regulation of diet, discipline as to going to stool at regular times, the avoidance of the habitual use of laxatives. This is especially important in families whose members manifest a tendency to constipation. Proper exercise is essential—walking, gymnastics, bicycle-riding, horseback-riding, swimming, etc., are excellent prophylactics.

2. *Dietetic Treatment.*—The proper selection of food is probably the most essential part of the treatment. A large number of cases will respond to a diet rich in residual constituents. It is my rule never to give instructions to my patients in a superficial way. I leave nothing to the judgment of my patients, but write out a full diet list, never using stereotyped, printed diet lists. The fact that you take interest in the case impresses the sufferer favorably and stimulates strict adherence to an explicit diet list.

For a patient suffering from atonic constipation, I give a diet list about as follows:

7 a.m., a glass of cold water, sometimes with the addition of a little salt or lemon juice. 8 a.m., oatmeal with cream, coffee with milk or cream, a tablespoonful of Merck's sugar of milk, eggs, graham bread with butter (no butter to obese people), about a tablespoonful of honey. After breakfast patient is directed to go to stool; no straining permitted.

1 p.m., different varieties of meat in moderation (except pork), no fried meats; plenty of all kinds of vegetables (even cabbage if digestion is good), lettuce, stewed apples and prunes, fruits fresh or preserved, graham bread and butter, and a glass of cider or buttermilk.

7 p.m., cold or warm meat (sparingly), mush and milk, graham bread and butter, fruit or jam.

In some cases with good appetite, I order a glass of buttermilk between meals, and at bedtime a saucerful of prune jam (i. e., stewed prunes passed through a sieve).

It will be seen from the above that the diet contains articles of food rich in residue, i. e., containing much cellulose. Honey, sugar of milk, cold water, buttermilk and cider are of great utility.

This holds good also for the *spastic* variety, but here the more digestible variety of vegetables and fruits must be given. Everything should be mashed or pureed. Too rough food would naturally increase the spasm and the catarrhal condition often associated with this form of constipation become aggravated.

3. *Mechanical Treatment.* — Patients with enteroptosis or splanchnoptosis should wear a well-fitting abdominal supporter. With massage, we can accomplish the most strikingly quick results, often after a few days' treatment, naturally always combined with the proper diet. It will pay every physician to learn to give gastro-intestinal massage to his patients himself. I never refer my cases to lay masseurs. The mode of administration is easy, one demonstration is sufficient to learn it. The fact that the physician himself takes the trouble to do the massage impresses the patient favorably and the result is better. I usually employ the different movements devised by Zabludowsky, of Berlin. Each sitting lasts from five to ten minutes. One daily treatment for one month, later on two or three time a week, according to results. Laxatives or purgatives are to be strictly prohibited. In the first week or two, a small enema of lukewarm water may be permitted every other day after breakfast.

In spastic constipation, massage seems to be contraindicated, as it increases intestinal spasm.

In these cases, the oil treatment as originally devised by Fleiner meets the indication. One-half tumblerful of warm olive oil, or sweet oil, is injected, at bedtime, through a soft rectal tube to which a glass funnel is attached. After the injection, I instruct the patient to lie on his stomach for ten or fifteen minutes. The oil is retained over night. In almost every case, a thorough evacuation occurs next morning. The oil dissolves the hard scybala and acts mechanically, stimulating peristaltic action. One injection every second or third day, in obstinate cases, is generally sufficient to effect a cure.

Gymnastics, home gymnastics, as taught by Schreber and Swedish gymnastics, are no doubt valuable adjuncts to the treatment of chronic constipation. Personally, I have had no experience with them, and so far I have been able to get along without these methods.

4. *Electricity.*—Electricity in obstinate cases is a most excellent remedial agent, especially in the atonic form of constipation. A strong foradic current should be employed, one plate electrode on the stomach, the other—one especially devised for such cases —is introduced into the rectum. Sitting five to ten minutes every second or third day.

5. *Hydrotherapy.* — In the absence of hydrotherapeutic institutes, where the treatment can be given scientifically, we can employ:

In atonic constipation, cold friction baths, douches, or full tub baths.

In the spastic forms, prolonged warm baths are serviceable. An alcohol or Priesnitz compress over the abdomen overnight is of decided benefit.

Europe has an abundance of watering places, where patients with chronic constipation are benefited and cured. We have a few in this country, but it is much to be regretted that patients do not get any systematic treatment, and are mostly left to their own discretion.

Tate Springs, French Lick, Saratoga waters act as purgatives as long as the waters are taken; as a rule, our patients relapse and are worse off from the weakened condition of the intestines to the excessive purging they subject themselves to.

6. Drugs will not cure any case of constipation; at best, they are palliative. A suppository of belladonna, or bellad, cautiously given with a little opium acts well in spastic constipation, relieving spasms and colicky pains.

In the vast majority of cases, a cure can be effected by diet alone or in combination with massage, and perhaps the oil treatment. This has been my experience, which, altogether, has been very gratifying.

Reference: Boas and Conheim.

DISCUSSION OF DR. AMSTER'S AND DR. TOEPEL'S PAPERS.

*Dr. J. B. Baird:* This is an important subject, from a practical standpoint, and I regret that the limited time allotted to it will not admit of its full discussion.

It would seem fair to assume that an individual living under physiological conditions would manifest no funtional derangement of the bowels. But experience teaches us that such is not the case. We find healthy infants at the breast, following literally nature's plan of diet and living, who suffer from obstinate constipation. The diet, so far as can be determined, can not be improved; more active exercise impracticable, regular habits and mental suggestion are not, in these cases, available influences, and can not be invoked. The physician can scarcely go every day to apply electricity or to employ massage. Daily visits to the office for the purpose of securing an action from the bowels would hardly be approved by the mother with household duties at home, or by the father, who is under the necessity of earning a living for his family; and yet we are in duty bound to find a solution of the problem. What advice shall we offer if the methods which have been advocated to-night can not, from force of circumstances, be successfully employed? Is it true that drugs are evil, and only evil, and evil continually in this malady? I think not. What shall we gain if we advise a commercial traveler to be regular in his habits, or select as to his diet, or to knead his abdomen for twenty minutes at a specified hour every morning? I suppose most of us have had to prescribe drugs after the so-called natural methods have failed.

Shall we gravely advise a bookkeeper who, in order to gain a livelihood for his family, toils at his desk all day, and possibly a part of the night, to take outdoor exercise and to live in the open air? We are not dealing with ideal cases, with ideal environment, or with ideal conditions. We must take our patients as we find them, and we are under obligations to give them practical and practicable advice, applicable to their necessities and circumstances, and based upon our knowledge and experience.

All of the measures advanced are serviceable; they may prove sufficient in some instances, and doubtless will help more or less in all. They may supplement, but they can never supplant drugs. I believe in drugs, I know that they are useful and that they give, in this complaint, immeasurable comfort when selected and applied with good judgment.

Vegetables and fruits, as a diet, are to be commended—but if they are not digested they fail in their laxative effect, and aggravate rather than alleviate constipation. Mechanical and local irritants may be used occasionally, but not habitually. Saline laxatives, on account of their hydragogue action, ultimately increase the trouble if constantly employed. Castor oil and cascara are useful drugs, so is rhubarb, and these are especially available in children, but the most satisfactory and most generally applicable drug for adults is aloes—in the form of the aqueous extract or the aloin. Small doses may be taken daily for years, if required, without increase, and with the greatest comfort and benefit. No one need fear becoming dependent upon this drug on account of its use. They are already dependent upon it, if they need it, before it is used. I fail to appreciate the wide difference between eating a quantity of fruit every day, with the chance of upsetting the stomach, and taking at bedtime a small mass of a vegetable substance divested of all extraneous, bulky and useless combinations. It seems to me that the effect is practically identical, and that the digestive organs, frequently not over strong, are thereby saved unprofitable, if not injurious, labor.

Let us use, if we can, the means proposed by the gentlemen who have read the interesting papers—regular habits, exercise, massage, a varied diet—not too concentrated, and all other natural forces—but also let us refrain from indiscriminately decrying drugs. We owe it to our patients and to our profession to use appropriate drugs at the right time and in the right way.

*Dr. Block:* It might be of interest to mention a class of cases which I have observed, where the feces are dry and hard, showing deficient moisture in the intestines, accompanied by the passage of large quantities of urine. Belladonna given three or four times a day, by lessening the urine excreted, has corrected the constipation by leaving more moisture to pass out through the intestines.

*Dr. Van Goidtsnoven:* My experience in the treatment of constipation is mostly amongst people who lead a sedentary life, ministers, lawyers, teachers, store-keepers, shop-girls, etc. This form of constipation seems to be, as a rule, associated with brain and physical disorders. Hence I always keep in view that the nervous system presides over all the functions of life, and a look over my prescription will show the *rationale* of its composition. I usually give:

℞ Pil. Hydrargyri:<br>

Fel. Bovis:<br>

Pil. Colocynth comp. . . . . . . . . . . . . . . . . . . . . . . . . . . . . . . . āā ʒss<br>

Pulv. Ipecacuanha . . . . . . . . . . . . . . . . . . . . . . . . . . . . . . . . . gr. vi<br>

Extr. Hyosiami sol . . . . . . . . . . . . . . . . . . . . . . . . . . . . . . . . . gr. xii<br>

M. Div. in Caps. xii.

S. One (1) capsule every night at bedtime. To be followed up next morning by a dose of Rochelle Salts in a goblet full of water.

*Dr. Amster* (closing): I am aware of the difficulties Dr. Baird mentions, which are encountered in treating cases of constipation, but this paper was not written for any special class, shop-girls, insurance men, drummers, etc. My object was to place before you a rational treatment, as I have found it to serve me in my own experience, and the experience of others. If I find a patient that can not employ any approved method, I simply do not care to treat his case or let him continue with his drugs. I know there are people who live to an old age, taking drugs to move their bowels, but that does not make it right and commendable to others. Dr. Baird claims there is no difference in taking an apple or a pill to relieve the constipation. I think any patient will prefer an apple or a rhubarb pie to pills or medicine, and then there is some nutritive value to an apple or a rhubarb pie.

I speak from my own experience when I say chronic constipation can be cured. For fifteen years I hardly ever went to bed without taking a pill to move my bowels. I knew I would not be fit for work the next day unless I resorted to some artificial means to move my bowels.

Two years ago I went to Europe, and was induced to give up drugs and eat more vegetables and fruits than I have been in the habit of eating. The result was instantaneous, and to this day I have not taken any laxative and am cured of my chronic constipation, having gained in the meantime twenty-five pounds.

